# Deep learning detects retropharyngeal edema on MRI in patients with acute neck infections

**DOI:** 10.1186/s41747-025-00599-6

**Published:** 2025-06-19

**Authors:** Oona Rainio, Heidi Huhtanen, Jari-Pekka Vierula, Janne Nurminen, Jaakko Heikkinen, Mikko Nyman, Riku Klén, Jussi Hirvonen

**Affiliations:** 1 Turku PET Centre, University of Turku and Turku University Hospital, Turku, Finland; 2 Department of Radiology, University of Turku and Turku University Hospital, Turku, Finland

**Keywords:** Artificial intelligence, Magnetic resonance imaging, Neural networks (computer), Respiratory tract infections, Retropharyngeal abscess

## Abstract

**Background:**

In acute neck infections, magnetic resonance imaging (MRI) shows retropharyngeal edema (RPE), which is a prognostic imaging biomarker for a severe course of illness. This study aimed to develop a deep learning-based algorithm for the automated detection of RPE.

**Methods:**

We developed a deep neural network consisting of two parts using axial T2-weighted water-only Dixon MRI images from 479 patients with acute neck infections annotated by radiologists at both slice and patient levels. First, a convolutional neural network (CNN) classified individual slices; second, an algorithm classified patients based on a stack of slices. Model performance was compared with the radiologists’ assessment as a reference standard. Accuracy, sensitivity, specificity, and area under receiver operating characteristic curve (AUROC) were calculated. The proposed CNN was compared with InceptionV3, and the patient-level classification algorithm was compared with traditional machine learning models.

**Results:**

Of the 479 patients, 244 (51%) were positive and 235 (49%) negative for RPE. Our model achieved accuracy, sensitivity, specificity, and AUROC of 94.6%, 83.3%, 96.2%, and 94.1% at the slice level, and 87.4%, 86.5%, 88.2%, and 94.8% at the patient level, respectively. The proposed CNN was faster than InceptionV3 but equally accurate. Our patient classification algorithm outperformed traditional machine learning models.

**Conclusion:**

A deep learning model, based on weakly annotated data and computationally manageable training, achieved high accuracy for automatically detecting RPE on MRI in patients with acute neck infections.

**Relevance statement:**

Our automated method for detecting relevant MRI findings was efficiently trained and might be easily deployed in practice to study clinical applicability. This approach might improve early detection of patients at high risk for a severe course of acute neck infections.

**Key Points:**

- Deep learning automatically detected retropharyngeal edema on MRI in acute neck infections.
- Areas under the receiver operating characteristic curve were 94.1% at the slice level and 94.8% at the patient level.
- The proposed convolutional neural network was lightweight and required only weakly annotated data.

**Graphical Abstract:**

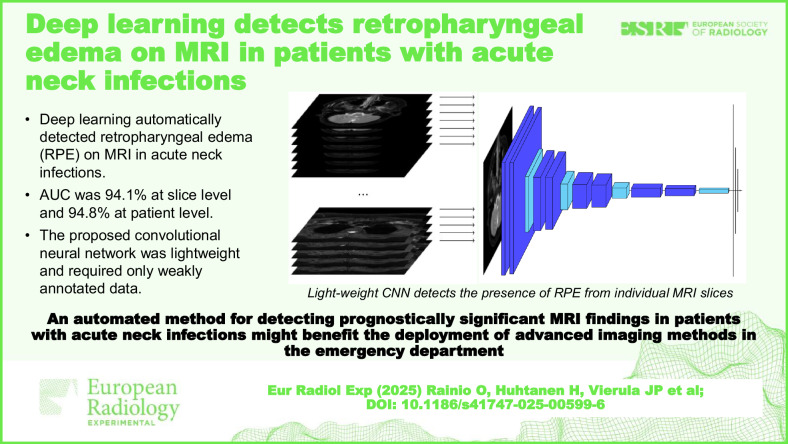

## Background

Acute neck infections require prompt diagnosis and intervention to prevent complications, especially when they reach the deep neck spaces [1]. Therefore, emergency imaging is critical in the early assessment of these patients [2]. Magnetic resonance imaging (MRI) has recently been validated as an effective primary diagnostic modality for imaging acute neck infections of various etiologies [3]. MRI has excellent soft tissue contrast and can offer improved diagnostic precision compared to the traditionally employed computed tomography. Previous research has demonstrated that emergency MRI is feasible in the acute setting [4, 5] and can accurately delineate abscesses and show edema patterns with high sensitivity [4, 6–9].

**Fig. 1:**
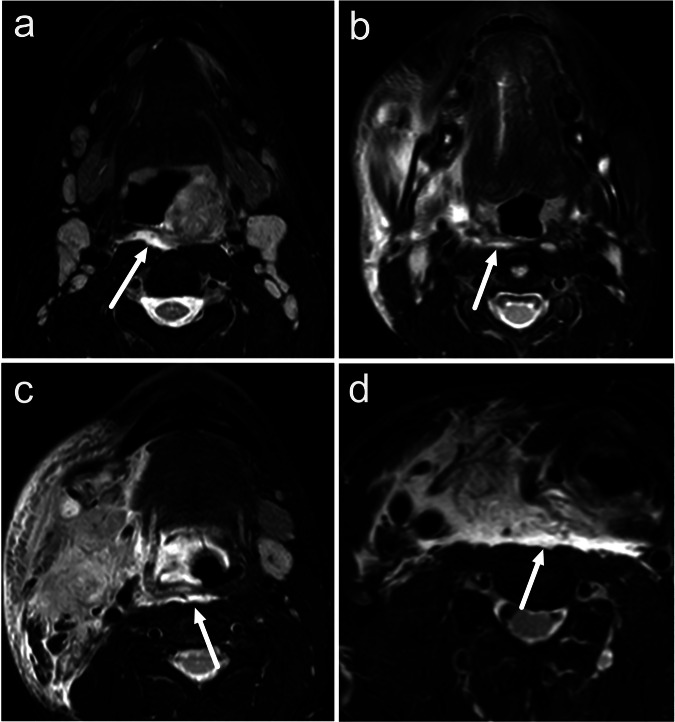
Examples of retropharyngeal edema (arrows) on axial T2-weighted Dixon water-only images of four different patients (**a**, **b**, **c**, **d**) with acute neck infections

About half of the patients with an acute neck infection show a hyperintense signal in the retropharyngeal space on MRI fluid-sensitive, fat-suppressed T2-weighted imaging, called retropharyngeal edema (RPE) [6] (Fig. 1). RPE is not a drainable fluid collection but rather a reactive edema pattern suggesting a severe course of illness. A previous study found that patients with RPE are much more likely to require admission to the intensive care unit than those without, suggesting that RPE could serve as an imaging biomarker for a severe course of illness [6]. This edema pattern has demonstrated substantial interobserver agreement between radiologists, suggesting favorable generalizability. However, considering the complex anatomy of the neck, variability in MRI sequences, and the subjective nature of this MRI-derived prognostic biomarker, the broader clinical applicability in the emergency setting would benefit from an automated method.

Deep learning (DL)-based artificial intelligence methods are commonly used for classification tasks in medical imaging [10] and would be particularly beneficial in an acute diagnostic setting concerning complex anatomy and advanced imaging [11]. Previous studies have successfully applied DL methods for the automated detection and segmentation of bone marrow edema on MRI in the sacroiliac [12–16], hip [17], and knee [18] joints and the spine [19], but an algorithm for detecting edema in the neck soft tissues is currently lacking. Here, we sought to develop and validate a DL algorithm for automated detection of RPE in fat-suppressed (water-only) T2-weighted MRI images from patients with acute neck infections.

## Methods

**Fig. 2:**
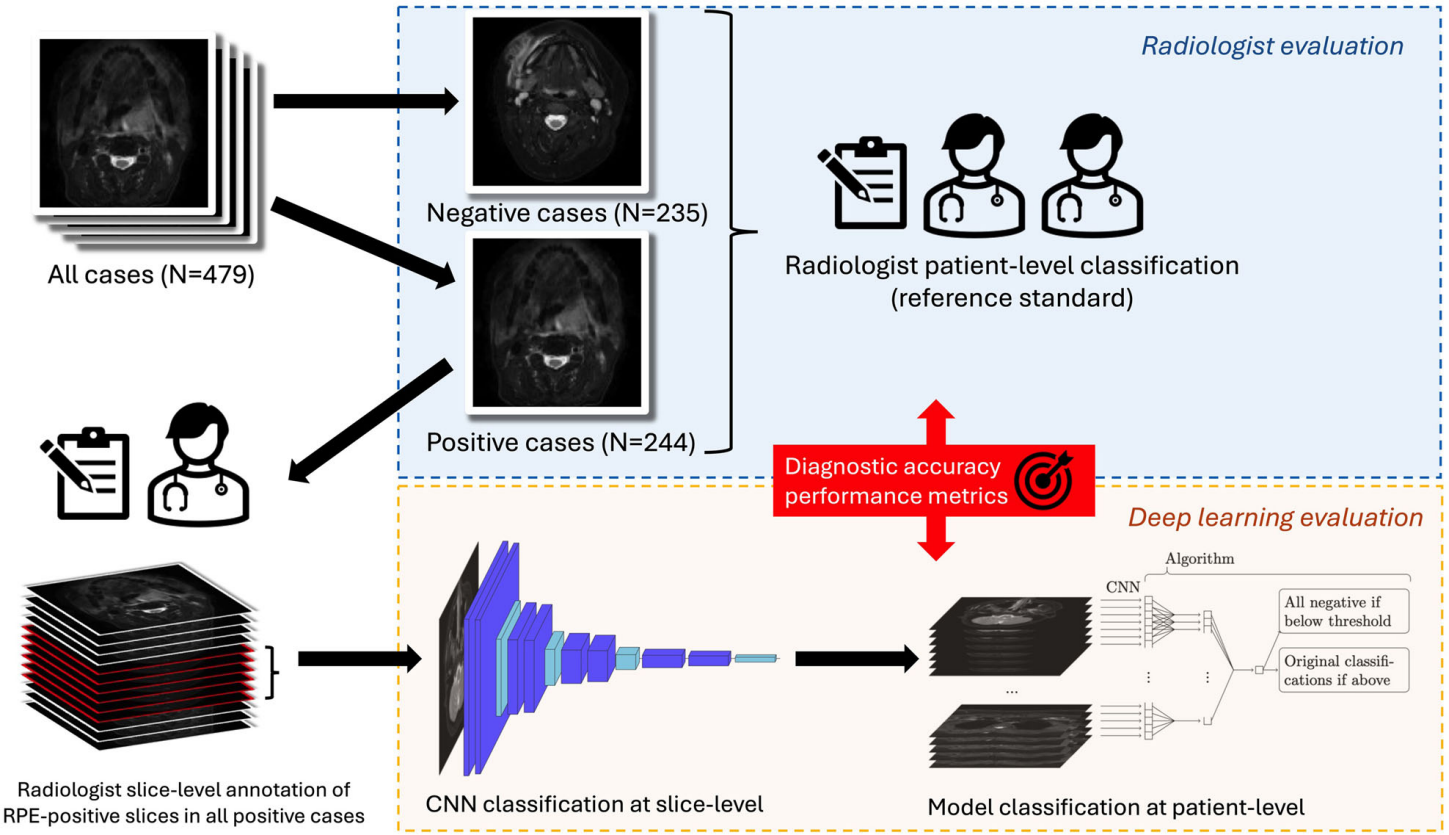
Study overview

The overall study workflow is presented in Fig. 2.

### Software and hardware

The image data was viewed and annotated with Carimas (version: 2.10) [20] and Mango (version 4.1, https://mangoviewer.com/). The experiments were performed with Python (version: 3.12.8) [21], using packages Keras (version: 3.7.0) [22], Tensorflow (version: 2.18.0) [23], sklearn (version: 1.5.2) [24] and SciPy (version: 1.14.1) [25]. No external computing services were used due to the sensitive nature of the patient imaging data. To obtain comparable estimates of the training time, all experiments were run on the same computer with Intel Core Ultra 5 125H processor and 16GB random-access memory.

### Data

This study followed previously published procedures for patient selection, MRI acquisition and interpretation, and the extraction of medical and surgical information [4, 6]. We obtained study permission from the hospital district board for this retrospective cohort study. The requirement of patient consent was waived due to the retrospective nature of the study. The inclusion criteria were: (1) emergency MRI between April 1, 2013, and August 30, 2021, for suspected neck infection scanned with the Philips Ingenia 3-T system using a dS HeadNeckSpine coil configuration; (2) MRI evidence of infection, that is, high signal on fat-suppressed T2-weighted Dixon images suggesting edema or high signal on fat-suppressed contrast-enhanced T1-weighted Dixon images suggesting abnormal tissue enhancement; (3) a final clinical diagnosis of an acute neck infection; and (4) available axial T2-weighted water-only Dixon images with diagnostic image quality as determined by a senior radiologist. The exclusion criterion was a lack of clinical and/or surgical data.

RPE was defined as an area of a hyperintense signal between the superior pharyngeal constrictor muscle anteriorly and the prevertebral muscles posteriorly on axial T2-weighted Dixon water-only images [6] (see Fig. 1). At the patient level, a consensus of two fellowship-trained neuroradiologists with significant experience and competence in emergency neck MRI formed the reference standard for RPE.

The final sample included 479 patients (277 males, 202 females), among whom 244 (51%) were positive and 235 (49%) negative for RPE. The mean age was 41 years (range 0–88 years).

Each MRI image contained 26–60 transaxial slices with a distance of 4–6 mm between the slices. The slices were all square-shaped images with pixel dimensions varying from 256 × 256 to 704 × 704. The pixels were between 0.342 × 0.342 mm^2^ and 0.781 × 0.781 mm^2^ in size, so that the real size of the transaxial slice varied from 16.0 × 16.0 cm^2^ to 25.0 × 25.0 cm^2^.

When training different convolutional neural network (CNN) set-ups for classification, each transaxial slice was considered as one data instance that should be classified either as positive or as negative. A fellowship-trained head and neck radiologist with significant experience in emergency neck MRI annotated the transaxial slices showing RPE in the positive patients by saving the indices of the uppermost and lowermost slices that showed RPE. By considering all the slices between these two indexes as positive and the rest as negative, we obtained 2,704 positive slices and 8,493 negative slices from the 244 positive patients. We gained an additional 10,830 negative slices from the 235 negative patients. Consequently, our total data contained 22,027 slices, 2,704 (12.3%) of which were positive.

All the slices were scaled from their original size to 128 * 128 pixels. They were normalized slice-wise separately by mapping their pixel values onto the interval [0,1] with

$${x}_{ij}- > ({x}_{ij}-\,min x)/(max x-\,min x)$$

where *max x* and *min x* are the maximum and the minimum of the current slice and *i*, *j* = 1,…,128. Due to memory restraints, the image arrays were converted from Python’s default float64 type to float32.

### Convolutional neural networks

The CNN of our proposed method is a modified U-Net encoder studied for head and neck cancer classification in an earlier study by Hellström et al [26]. The original U-Net introduced in 2015 by Ronneberger et al [27] was designed for medical image segmentation. It consists of two paths, a contracting one and an expanding one, the former of which is also called an encoder and the latter a decoder. The idea behind this encoder-decoder design is that the encoder first shrinks the image in size to be able to see the context of the whole image, and the decoder returns the image to the original size so that it can focus on the details needed to perform accurate segmentation. Additionally, the original U-Net also copies the data inputs at different stages of the encoder and sends them to the decoder over several layers to prevent the loss of information during the shrinking process. Both the encoder and the decoder contain sequences of paired convolutions followed by max-pooling operations.

**Fig. 3:**
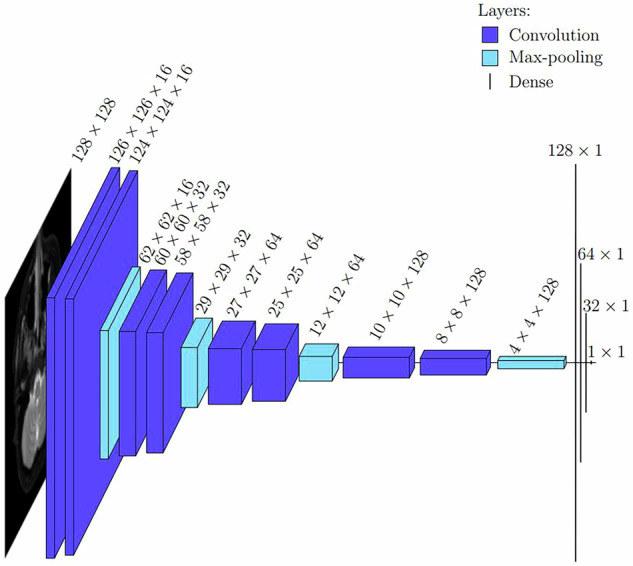
The architecture of our convolutional neural network with all the layers and their dimensions visible

However, it was noted by Hellström et al [26] that the U-Net encoder can be used to create an effective lightweight CNN for classification. Namely, U-Net is able to detect relevant patterns on the input data during the encoder, and if the aim is to obtain a single class label, there is no need for the decoder. Instead, only a few dense layers are added after the encoder, as shown in Fig. 3. Given this new design worked for head and neck cancer classification, we assumed that it might be well-suited for the current classification task, too, even if our data is MRI images instead of positron emission tomography images used by Hellström et al [26].

The CNN based on the U-Net encoder was compared to InceptionV3, which is considered a state-of-the-art CNN for medical image classification [28]. Introduced by Szegedy et al [29], it is an improvement of the earlier Inception networks that utilize several differently sized kernels [30]. As InceptionV3 has three color channels, it was given three identical copies of each original grayscale image matrix stacked on top of each other as inputs. While InceptionV3 can be downloaded with weights pre-trained on ImageNet dataset to apply transfer learning, we did not use these pre-trained weights because the colored photographs of ImageNet differ from our grayscale MRI data enough to prevent a fair comparison between the proposed CNN and InceptionV3.

The CNN set-ups were trained with repeated five-fold cross-validation so that we first divided the data into five folds of around 20% of the total data and then each one of the original five folds was used as the test set and the other four as training data for six times, meaning 30 training iterations in total. The five-fold data split was done patient-wise, and the CNN was re-initialized after each iteration. Additionally, during each training iteration, we randomly chose a subset of 30% from the training data to create a validation set so that we could monitor the generalizability of the learning and define an early stopping criterion. The validation data was not used for any other purposes. Consequently, in each iteration, 20% of the total data was used for testing and, from the remaining 80% data, 70% (56% of the total data) was used for the actual training and 30% (24% of the total data) for validation. The loss function was the standard binary cross-entropy, which is compatible with the sigmoid activation used on the last layers of our proposed CNN and InceptionV3 implementations. Adam was chosen as an optimizer because it was noted that the convergence of the binary cross-entropy was very slow and uneven with the standard stochastic gradient descent. Based on a few initial tests, both the proposed CNN and InceptionV3 obtained an accuracy over 90% in the training set during the first two or three epochs, with very little stepwise progress afterwards. Consequently, the number of epochs was set as 15, but early stopping was used if no improvement was obtained in the validation loss during five consecutive epochs. The training time was recorded for each iteration.

#### Data augmentation

In our proposed method, the number of training images is doubled by including their reflections over the vertical axis in the training data. This type of augmentation was compared to the results obtained with no augmentation and augmentation based on clockwise rotation of 90 degrees and blurring images along filters of 3 × 3 pixels. These three specific types of augmentation were originally chosen for this study because it was observed in the article [31] that a reflection in a vertical direction, a rotation of 90 degrees, and a Gaussian blur worked the best with MRI datasets.

### Post-processing algorithm

The numeric predictions given as an output for the test set by a CNN are typically converted into binary labels by using the threshold that maximizes the Youden’s index for the predictions of the training set [32]. The Youden’s index is defined as the sum of sensitivity (the percentage of the positive instances classified correctly) and specificity (the percentage of the negative instances classified correctly) minus 1 [33]. After this, all the patients with at least one slice predicted as positive can be classified as positive and the rest of the patients as negative. However, this approach likely results in a very low patient-wise specificity. Namely, even if the specificity among the slices would be very high, even a single false positive prediction among all the 26–60 slices of a negative patient would cause a false positive prediction for the given patient. To avoid this issue, we need to first use the numeric predictions of the slices to classify the patients and, only after that, classify the slices in a way that all the slices of the negative patients are negative.

**Fig. 4:**
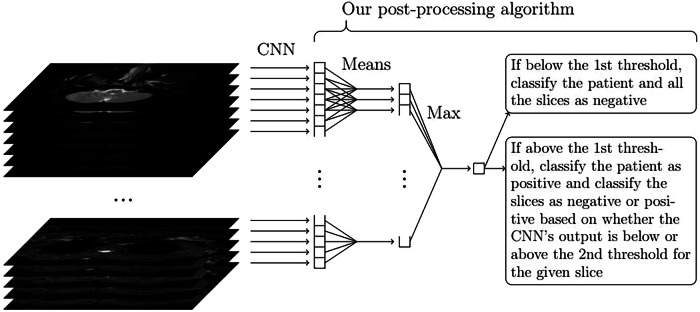
The structure of our proposed method to obtain the final classifications of a single patient: First, the convolutional neural network (CNN) is used to obtain numeric slice-wise predictions, then the maximum of the mean values of five consecutive slice-wise predictions is computed, and the patient-wise classification is obtained based on whether the maximum is below or above a certain threshold. If the patient is classified as positive, the slice-wise predictions are converted into binary labels with a second threshold; otherwise, they are all classified as negative. The two thresholds are chosen by finding the thresholds that give the maximal Youden’s index for the patient- and slice-wise predictions with this same method for the training set predictions. Our post-processing algorithm finds the appropriate thresholds and converts the predictions of all the test set patients into final binary labels

Consequently, in our proposed approach, we perform the post-processing scheme as in Fig. 4 with a new algorithm specifically designed by us for this purpose. As input, this algorithm receives all the numeric slice-wise predictions obtained by the given CNN set-up from both the training and the test set, ordered as vectors so that each vector contains all the slice-wise predictions of a single patient in the correct order. Additionally, the algorithm receives information about the correct classifications of the training set patients. It then goes through the vectors of the test set, lists the mean values of each set of five consecutive slice-wise predictions, and chooses the maximum of these mean values for each vector. The number five was used here because we needed both information from at least a few adjacent slices, but still a number small enough not to lose sensitivity in the detection of RPE visible only in 2–3 slices. The patient is classified as positive or negative based on whether this maximum is above or below a threshold value, which is equal to the threshold that gives the maximal Youden’s index among the predictions of the training set with this exact same approach. After this algorithm, the final predictions of the test set slices were obtained by interpreting all the slices from such patients that were classified as negative by the algorithm as negative and converting the rest of the slice-wise predictions as positive or negative according to the maximal Youden’s index chosen based on the training data.

This new algorithm was compared with both the Random Forest and the Support Vector Machine (SVM) algorithms. They were trained by using similar patient-wise vectors and the correct labels of the patients within the training set, though these vectors were padded with additional zeros to obtain the same dimension for all the inputs. The predictions of the test set vectors were converted into binary labels by using the maximal Youden’s index of the training set vectors.

### Evaluation

To evaluate our proposed method, we computed the values of accuracy, sensitivity, specificity, and the area under the receiver operating characteristic curve (AUROC) [32] on both patient level and slice level for the predictions of each test set during the repeated cross-validation. The AUROC of the proposed method on the patient level was obtained by considering the curve drawn by using different thresholds to convert the patients based on the maximums for the mean values of five consecutive slice-wise predictions. Additionally, we compared the proposed CNN and vertical reflection-based augmentation to InceptionV3 and other augmentation choices by computing the same four evaluation metrics directly from the slice-wise CNN output of the test set, with no patient-wise post-processing algorithm. We also assessed the training times of the CNN setups. The comparison between our new post-processing algorithm, random forest, and SVM was done with the same four evaluation metrics on the patient level. As all the CNN setups are trained with five-fold cross-validation repeated six times, we obtained 30 different values for each method variation for the purpose of statistical testing.

### Statistical analysis

To study whether there were significant differences between 30 different values of accuracy, sensitivity, specificity, or AUROC, we used the Wilcoxon signed-rank test. The reason for this test is that it is non-parametric and therefore not sensitive to potential outliers, and its assumption of paired observations suits our situation where the cross-validation test set varies over different iteration rounds [32]. The multiple comparisons problem was taken into account before drawing conclusions by considering only the *p*-values less than 0.01 or 0.001 significant instead of using the standard level of 5%.

## Results

**Fig. 5:**
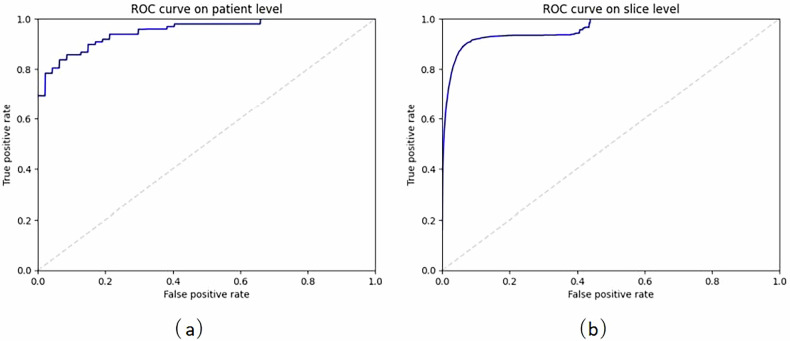
The median AUROC curves on (**a**) patient and (**b**) slice level computed from the medians of the ROC curves of the test set predictions by our proposed method after each 30 training iterations

**Fig. 6:**
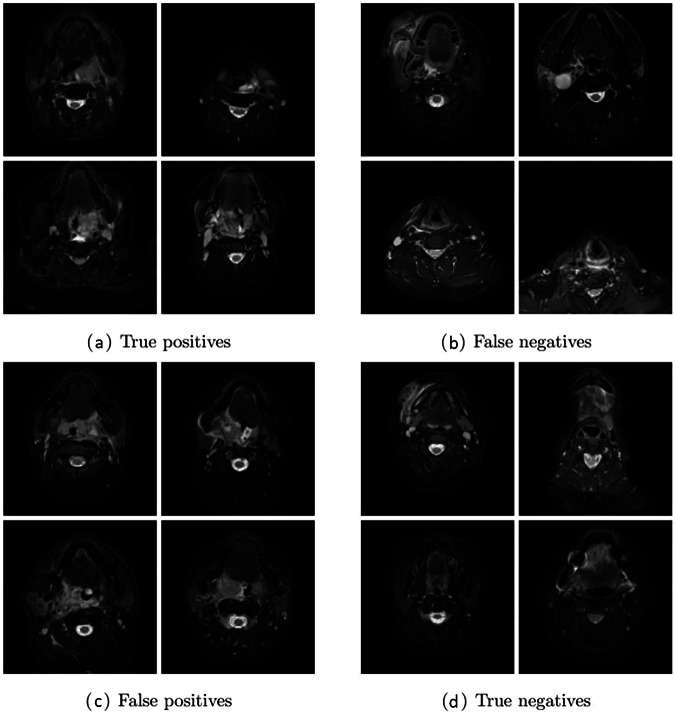
Examples of 16 slices of the first test set that were consistently classified either correctly or incorrectly during the 6 repetitions of the first five-fold cross-validation split, including (**a**) positive slices correctly classified as positive, (**b**) positive slices incorrectly classified as negative, (**c**) negative slices incorrectly classified as positive, and (**d**) negative slices correctly classified as negative by our proposed method. All the slices are from different patients

Our proposed method is the CNN based on the U-Net encoder with vertical reflection augmentation, followed by our new algorithm, the results of which are summarized in Table 1. Due to the large number of negative slices, the method has a very high slice-wise specificity, but it is also accurate and sensitive on both the patient and slice levels. In particular, the AUROC values are very high, meaning that the method should work relatively well on different choices of thresholds used for converting numeric predictions. Figure 5 shows the median ROC curves corresponding to the AUROC values of Table 1, and Fig. 6 shows examples of the correct and the incorrect classification decisions by our proposed method.

**Table 1:** Evaluation metrics for the proposed CNN

Metric	Patient level	Slice level
Accuracy (%)	87.4 ± 2.8/87.0	94.6 ± 0.4/94.6
Sensitivity (%)	86.5 ± 3.2/85.7	83.3 ± 3.6/82.7
Specificity (%)	88.2 ± 4.9/87.2	96.2 ± 0.7/96.2
AUROC (%)	94.8 ± 1.2/94.9	94.1 ± 1.3/94.0

Data are given as mean ± standard deviation/median of the four evaluation metrics computed both on patient level and on slice level with our proposed method (the convolutional neural network shown in Fig. 3 with vertical reflection augmentation followed by our new algorithm) during the 30 iterations of the repeated five-fold cross-validation

Tables 2 and 3 show the results of the comparison between the proposed CNN and InceptionV3 and between different augmentation choices, respectively. As can be seen from Table 2, even though the proposed CNN and InceptionV3 perform very similarly otherwise, the proposed CNN is considerably more computationally effective: The training period of the non-augmented CNN is less than 10 min, whereas training InceptionV3 takes over 4 h with the same data and hardware. Table 3 reveals that the vertical reflection produces significantly better AUROC values than the other augmentation choices, even if no augmentation method is superior in terms of all four evaluation metrics due to the trade-off between sensitivity and specificity.

**Table 2:** The proposed CNN *versus* InceptionV3

Metric	Proposed convolutional neural network	InceptionV3
Accuracy (%)	**91.6** ± **0.9**/91.5	91.6 ± 1.1/**91.7**
Sensitivity (%)	**89.3** ± **2.4/89.2**	89.2 ± 2.6/89.1
Specificity (%)	**91.9** ± **1.2/91.8**	91.9 ± 1.5/92.1
AUROC (%)	**96.9** ± **0.5/97.0**	96.7 ± 0.7/96.5*
Time (min)	**9.3** ± **1.1/9.1**	277.7 ± 60.5/270.1***

Data are given as mean ± standard deviation/median of the four evaluation metrics for the predictions of the test set slices and the training time of a single iteration for the proposed convolutional neural network and InceptionV3 during the 30 iterations of the repeated five-fold cross-validation. No augmentation nor post-processing algorithm was used. The values of InceptionV3 that significantly differ those of the proposed convolutional neural network according to a Wilcoxon signed-rank test are denoted as follows: *p < 0.05; **p < 0.01; ***p < 0.001. The better values of evaluation metrics and time are in bold

**Table 3:** Impact of augmentation

Metric	Reflection	No augmentation	Rotation	Blurring
Accuracy (%)	92.8 ± 0.8/92.8	91.6 ± 0.9/91.5***	91.5 ± 1.2/91.6***	**92.8** ± **0.7/92.8**
Sensitivity (%)	89.0 ± 2.8/88.5	89.3 ± 2.4/89.2	**90.3** ± 2.2/90.2*	81.4 ± 3.6/81.1***
Specificity (%)	93.4 ± 1.1/93.3	91.9 ± 1.2/91.8***	91.7 ± 1.6/91.8***	**94.4** ± 1.0/94.6**
AUROC (%)	**97.3** ± **0.4/97.3**	96.9 ± 0.5/97.0***	97.1 ± 0.3/97.0**	96.3 ± 0.6/96.3***
Time (min)	22.2 ± 2.6/22.0	**9.3** ± 1.1/9.1***	19.6 ± 2.3/19.3**	31.9 ± 3.2/32.7***

Data are given mean ± standard deviation/median of the four evaluation metrics for the predictions of the test set slices and the training time of a single iteration for the proposed convolutional neural network with different augmentation choices during the 30 iterations of the repeated five-fold cross-validation. These choices include either augmentation with vertical reflection, no augmentation, augmentation with 90-degree rotation, or augmentation with image blurring, and each augmentation type was used to double the amount of the original training data. No post-processing algorithm was used. The best values are in bold and the values that significantly differ from those of the vertical reflection augmentation according to a Wilcoxon signed-rank test are denoted by *, **, and *** as in Table 2

Table 4 summarizes the patient-wise predictions obtained by our new algorithm compared to random forest and SVM. The predictions are all computed by using the numeric output of the proposed CNN with vertical reflection augmentation. As can be seen, our new algorithm has the highest accuracy and AUROC, and it outperforms the two other algorithms in terms of AUROC in a statistically significant way.

**Table 4:** Proposed post-processing algorithm *versus* Random forest and SVM

Metric	Our algorithm	Random forest	Support vector machine
Accuracy (%)	**87.4** ± **2.8/87.0**	85.9 ± 2.7/86.4***	86.8 ± 3.1/86.5
Sensitivity (%)	86.5 ± 3.2/85.7	**87.3** ± **3.3/87.5**	85.7 ± 3.8/85.7
Specificity	**88.2** ± **4.9/87.2**	84.5 ± 4.5/85.1***	87.9 ± 4.3/88.3
AUROC (%)	**94.8** ± **1.2/94.9**	86.1 ± 2.6/86.4***	86.8 ± 3.1/86.5***

Data are given as mean ± standard deviation/median of the four evaluation metrics for the predicted classifications of the test set patients computed with our new algorithm, random forest, and support vector machine from the slice-wise predictions obtained with the proposed convolutional neural network with vertical reflection augmentation during the 30 iterations of the repeated five-fold cross-validation. The best values are in bold and the values that significantly differ those of our new algorithm according to a Wilcoxon signed-rank test are denoted by *, **, and *** as in Table 2

## Discussion

In emergency MRI of acute neck infections, RPE is an imaging biomarker with clinical and prognostic significance. Here, our DL-based algorithm for the automated detection of RPE from axial T2-weighted water-only Dixon images achieved high diagnostic accuracy on both slice and patient levels against the reference standard of a consensus between two neuroradiologists. Our model’s high accuracy and manageable computational requirements pave the way for clinical implementation in the emergency setting.

Our proposed method consists of a classifier CNN based on the encoder of the U-Net, combined with a simple algorithm. While the CNN is two-dimensional and processes the transaxial slices separately, our new algorithm allows us to utilize the depth-wise information of the original three-dimensional MRI images. Additionally, the training of our proposed CNN takes only 3.3% of the time required by the state-of-the-art classification CNN InceptionV3 without resulting in a less accurate classification performance. While the inference times were not recorded or compared between the models, they are around a few seconds for the whole test data, and their potential differences would, therefore, not have a practical meaning when predicting the image of a specific patient in the emergency department. Consequently, our method is both very lightweight and accurate. In addition, we used only weakly annotated data based on slice- and patient-based binary labels instead of annotating RPE specifically within positive slices.

The accuracy of our DL-based algorithm was higher at the slice level than at the patient level. Specificity (the proportion of true negatives to all negatives) dropped slightly more than sensitivity from the slice level to the patient level, most likely due to the considerably higher number of true negatives at the slice level (88%) than at the patient level (49%). Nevertheless, the accuracy at the patient level is reasonably promising for clinical applications, given that the pre-test probability of RPE is not particularly low (about 50%).

Despite overall high accuracy, some misclassifications were evident (Fig. 6). RPE is defined as high signal area between the superior constrictor muscle and the prevertebral muscle. Some false positives appear to have been due to edema (high signal intensity) in the superior constrictor muscle itself (Fig. 6c). In contrast, some false negatives are unilateral and thin appearances of RPE (Fig. 6b) that diagnostic radiologists have nevertheless classified as positive. These false model classifications, although rare, partially highlight the difficulties associated with the subjective nature of the reference standard.

Our findings suggest some directions for future research. Our approach involved a CNN prediction of the patients’ RPE status from individual axial slices. An alternative strategy would be to employ a three-dimensional CNN, although this would be computationally more burdensome. Another avenue for future work would be to use patient-level annotation and only the images as input to directly predict clinical outcomes, such as intensive care unit admissions. This would bypass the subjective reference standard of RPE, representing an operationalized imaging biomarker or a proxy for human observers. Such direct classification might have higher accuracy for specific clinical outcomes. Still, it would inherently be limited to only one outcome at a time, unlike RPE, which is associated with multiple clinical and laboratory outcomes in acute neck infections [6] and thus perhaps more versatile.

Our algorithm might be clinically applied as an automated triage tool to prioritize neck MRI scans with clinically and prognostically significant findings, alerting the on-call radiologist. In addition, this algorithm might improve the diagnostic accuracy of radiologists who are less familiar with acute neck MRI interpretation during on-call hours. Finally, automated tools for image interpretation might be helpful for clinicians, who often view images themselves.

Particular strengths of the study include a large sample size of high-quality 3-T MRI images from patients with acute neck infections of various etiologies, corroborated by thorough clinical and surgical characterization. From the technical and generalizability perspective, a considerable strength is the high performance of the model with very manageable hardware requirements. Yet, some limitations need to be addressed. The most critical limitation is the subjective nature of the reference standard for RPE, based on the consensus of two experienced and fellowship-trained head and neck radiologists. This limitation is inherent in many studies on semiquantitative imaging biomarkers, such as bone marrow edema in sacroiliitis [12]. Next steps to further corroborate this imaging finding might be to establish the intra- and interobserver reproducibility in a large sample of annotators with varying levels of expertise and to grade RPE using automated volumetric segmentations to achieve more fine-grained correlations to patient outcomes. Related to the reference standard, perfect model performance would be as good as that of a consensus of two fellowship-trained neuroradiologists. A practical implication is that the model would benefit radiologists and clinicians who are less familiar with acute neck MRI interpretation. Yet, this is a probable setting in most emergency radiology departments, especially outside office hours. All MRI data were obtained from a single institution, excluding external site validation and limiting generalizability. We are unaware of other large published MRI datasets on acute neck infections. In general, the availability of emergency MRI is much lower than that of computed tomography, the most commonly used cross-sectional imaging method in acute neck infections. This will limit potential clinical applicability, which will be required to assess the impact on reporting times and patient outcomes. Concerning the DL algorithm, we could not produce attention maps, limiting the explainability of our model. In any case, the high performance of the model should be useful in clinical practice.

In conclusion, we found sensitivity, specificity, and AUROC of 86.5%, 88.2%, and 94.8%, respectively, for a DL-based algorithm for automated detection of RPE, an MRI imaging biomarker of clinical and prognostic significance in patients with acute neck infections. Highly accurate automated methods for patient prognostication may improve patient outcomes and the generalizability of advanced imaging methods in the emergency setting.

## Data Availability

Data cannot be publicly shared because of the national legislature’s on the privacy of patient data. The code is available at https://github.com/rklen/Rpe_project.

## Abbreviations

AUROC: Area under receiver operating characteristic curve
CNN: Convolutional neural network
DL: Deep learning
MRI: Magnetic resonance imaging
RPE: Retropharyngeal edema
SVM: Support vector machine
